# Combination of Autophagy Selective Therapeutics With Doxil: An Assessment of Pathological Toxicity

**DOI:** 10.3389/ftox.2022.937150

**Published:** 2022-06-29

**Authors:** Kristi L. Helke, Radhika R. Gudi, Chenthamarakshan Vasu, Joe R. Delaney

**Affiliations:** ^1^ Departments of Comparative Medicine, and Pathology and Laboratory Medicine, Medical University of South Carolina, Charleston, SC, United States; ^2^ Department of Microbiology and Immunology, Medical University of South Carolina, Charleston, SC, United States; ^3^ Department of Biochemistry and Molecular Biology, Medical University of South Carolina, Charleston, SC, United States

**Keywords:** autophagy, chemotherapy, doxorubicin, hydroxychloroquine (chloroquine), nelfinavir, histopathology

## Abstract

**Background:** Combination therapy of targeted drugs in cancer treatment is a field in constant flux, with research balancing side effects with efficacy. Efficacy from combination therapy is improved either through synthetic lethality or through prevention of recurrent clones. Previous research has shown (hydroxy-)chloroquine is insufficient to disrupt autophagy in tumors. Hence, either combinations or novel autophagy agents are desired. *In vivo* studies of ovarian cancer have revealed that chloroquine can be combined with up to four other autophagy drugs to suppress ovarian cancer growth. While cancer efficacy is now established for the autophagy drug combination, it is unclear what toxicities may require monitoring in human trials. Additive toxicity with chemotherapy is also unknown.

**Methods:** To address toxicity in more depth than previous weight-monitoring studies, biochemical and histopathology studies were performed. Mouse groups were treated with autophagy drugs for 2 weeks, with or without the chemotherapy Doxil. After the last dose, mice were processed for blood biochemistry, white blood cell markers, and histopathology.

**Results:** Data from a comprehensive blood biochemistry panel, flow cytometric measurements of blood cell markers, and histopathology are herein reported. While Doxil presented clear bone marrow and immunologic toxicity, autophagy drugs were overall less toxic and more variable in their presentation of potential toxicities. Only minor additive effects of autophagy drugs with Doxil were observed.

**Conclusion:** Combinations of autophagy drugs may be considered for therapy in human oncology trials, with possible side effects to monitor informed by these murine pre-clinical data.

## Introduction

Autophagy is the mechanism cells use to clean house of misfolded proteins and debris *via* trafficking them to lysosomes. Autophagy modulating drugs have been tested in over one thousand clinical trials in cancer, but have had limited success due to poor autophagy engagement or dose-limiting toxicity (Duffy et al., 2015; Chude and Amaravadi, 2017; Levy et al., 2017; Amaravadi et al., 2019). As a macromolecule and organelle recycling system, autophagy’s fully competent and properly regulated flux in cancer cells is advantageous to tumor survival in stressed contexts. Literature suggests there may be two broad categories of autophagic vulnerability for cancer: cancers which are addicted to elevated autophagy and require autophagic flux for survival, and those cancers which suppress autophagy to promote genomic instability and initiate cancer (Levine and Kroemer, 2008; 2019). These two categories are not necessarily mutually exclusive; tumors which lose autophagy genes for tumorigenesis may nonetheless later require autophagic flux during hypoxia, chemotherapy, cancer stem cell survival, immune evasion, or other stresses (Kimmelman and White, 2017). Autophagy-addicted cancers have been well studied in the context of leukemia, *RAS*-mutant, and *BRAF* mutant tumors (Kimmelman and White, 2017; Poillet-Perez and White, 2019). We recently showed a correlation of suppressed autophagy with p53-mutant tumors (Bowers et al., 2022b), consistent with the role of p53 in causing apoptosis in low autophagy cells (Yang et al., 2020). Monoallelic loss of a commonly deleted autophagy gene *BECN1* has been validated as tumorigenic in breast cancer (Cicchini et al., 2014) and ovarian cancer (Delaney et al., 2020). We found evidence that single-cells within a *BECN1* ^+/-^ ovarian tumor are more variably aneuploid cell-to-cell than a similarly analyzed *BECN1* ^+/+^ tumor (Kumar et al., 2020). High-grade serous ovarian carcinoma (OV) contains more autophagy gene losses than other well-characterized solid tumors (Delaney et al., 2017; Bowers et al., 2022b).

One strategy to potentially improve the clinical success of autophagy modulating drugs in a cancer setting is to combine drugs which disrupt autophagy through different molecular mechanisms. Metformin **(M)** and rapamycin **(R)** are commonly used autophagy activators, which act *via* mitochondria complex I inhibition and AMPK activation (metformin), or by inhibition of mTORC1 (rapamycin). Chloroquine and its more tolerable analog hydroxychloroquine **(C)** have been used as autophagy inhibitors, working by disruption of autophagosome-lysosome fusion as well as Golgi dispersion (Mauthe et al., 2018). All three of these drugs have substantial pre-clinical evidence of slowing or halting tumor growth in both autophagy-elevated (Rosenfeldt et al., 2013; Kasznicki et al., 2014; Li et al., 2014) and autophagy-suppressed cancer types (Huynh et al., 2007; Shank et al., 2012; Delaney et al., 2015; Liu et al., 2015; Pagotto et al., 2017). We additionally identified dasatinib **(D)** as an autophagy activator (Delaney et al., 2015), predictably through inhibition of SRC and other tyrosine kinases upstream of autophagy initiation factors (Milano et al., 2009; Le et al., 2010). Nelfinavir mesylate **(N)** has been shown to contribute to endoplasmic reticulum stress (Pyrko et al., 2007), resulting in compensatory upregulation of autophagy. We discovered nelfinavir can additionally prevent proper autophagic flux (Delaney et al., 2017). By carefully studying pairwise and higher order combinations of these five drugs, we found none of the drugs offset the effects of any other autophagy drug (Delaney et al., 2017), despite some drugs being considered autophagy activators and some being considered autophagy inhibitors. The simplest explanation is there is no true “activator” of autophagy in these five drugs; all drugs increase autophagy markers because of a stress created within the cell, which autophagy is upregulated in response to. In a live-cell time course, we observed induction of autophagosome formation preceded cell lysis, but drug combinations resulted in earlier and earlier lysis the more toxic the combination was (Delaney et al., 2017).

Safety is always a concern in combinatorial therapies. We have previously demonstrated strong *in-vivo* efficacy of the Combination Of Autophagy Selective Therapeutics (COAST), as defined as containing a combination of the five drugs enumerated here (M, C, N, R, and/or D). Subcutaneous, syngeneic, and patient-derived xenografts of OV models were suppressed in tumor growth using all five drugs (Delaney et al., 2017). The syngeneic ID8IP-mCherry model revealed residual microscopic tumor when using the most cytotoxic pair, C and N, suggesting three drugs or more may be most efficacious; two drugs may not be enough to remove residual disease. This combination of five drugs is currently being investigated in a human clinical trial (NCT05036226). Two important questions remained, which could be addressed in preclinical models to better inform future human trials. First, are there any predictable side effects from combining these five autophagy drugs? Second, since drugs are often combined with second-line chemotherapy in initial efficacy-testing clinical trials, do side effects worsen when combined with such a second-line chemotherapy? To address these two questions in a single set of well-controlled, highly-monitored mice, we chose a chemotherapy model of peggylated liposomal doxorubicin, **Doxil**, a second-line chemotherapy in OV. We tested for drug-induced toxicity in pre-clinical mouse models and report here all histological and biochemical phenotypes observed, far surpassing the typical toxicity measure of simple weight-loss. These results are intended to better inform design of future clinical trials utilizing autophagy drugs in combination.

## Materials and Methods

### Animals

**FIGURE 1:**
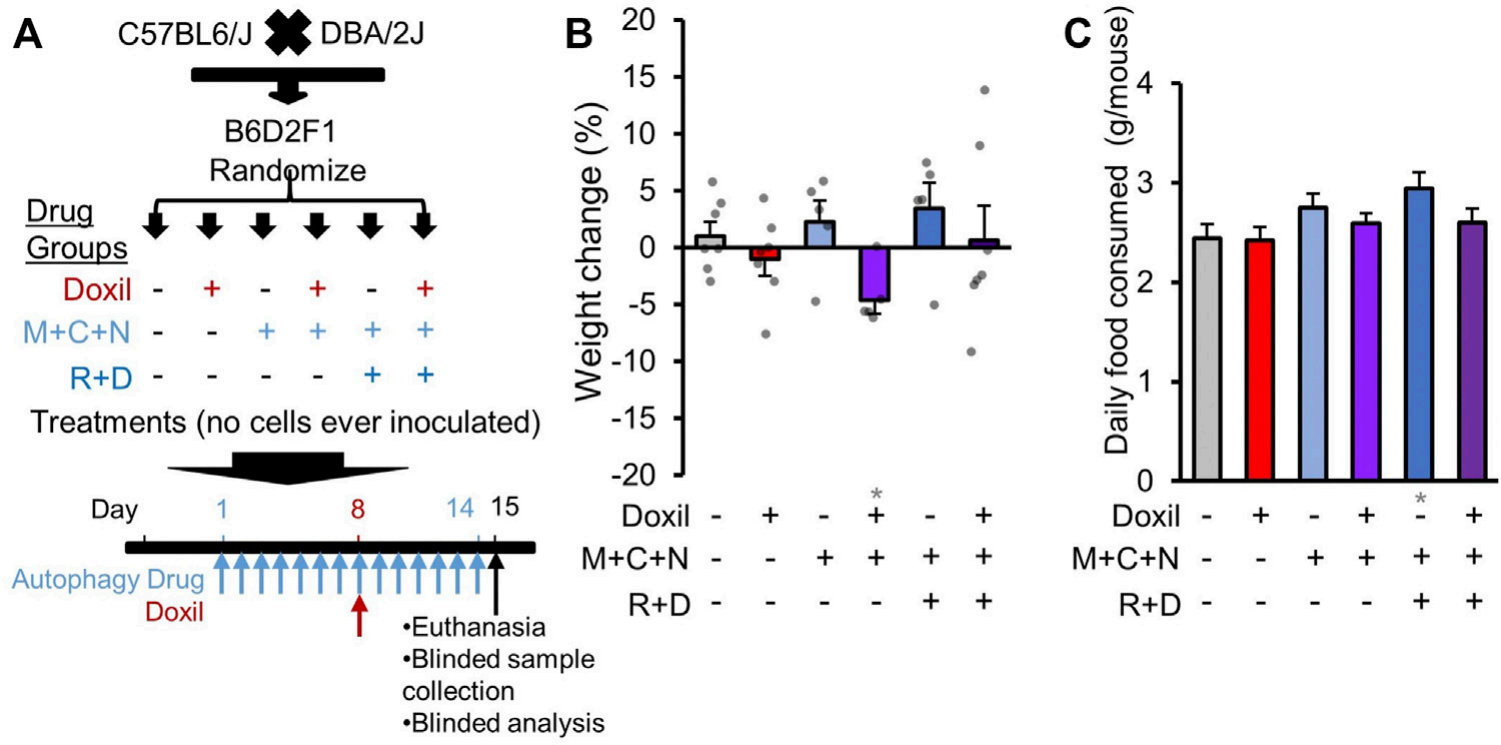
Study design and whole-organism safety measures. **(A)**. Diagram of experimental design. B6D2F1 hybrid mice were obtained by crossing C57BL6 and DBA/2 mice. No cancer cells were inoculated. Since the diseases of interest for combined therapy with Doxil are gynecologic cancers, only female mice were assayed. Once mice reached 8-weeks of age, mice were randomized into six treatment groups. Autophagy groups included (i) vehicle control (226 mg/kg microcrystalline cellulose in 50% PEG400), (ii) “M + C + N” indicating metformin (M, 205 mg/kg/day) + hydroxychloroquine (C, 100 mg/kg/day) + nelfinavir mesylate (N, 250 mg/kg/day), or (iii) “R + D” group which contained M + C + N and additionally rapamycin (R, 2.24 mg/kg/day) + dasatinib (D, 10 mg/kg/day). All autophagy class drugs were administered by gavage, daily for 14 days (see Methods). Chemotherapy groups included (a) vehicle control (0.9% sterile saline solution) or (b) Doxil (15 mg/kg) administered through intraperitoneal injection on day 8 with 200 µL weight-adjusted maximal volume. Tissues and blood were extracted for analysis on day 15, as depicted. **(B)**. Mouse weight was assessed on the day of euthanasia and compared to baseline as a percent change. Bars indicate means ± SEM. and individual mice are represented as overlaid dots. *P* was >0.05 for all groups relative to vehicle control. **(C)**. The amount of food eaten by each cage of mice was averaged over a period of 11 days and divided by N = 5 mice. Gray * indicates *p* ≤ 0.05 relative to vehicle control.

Female mice were purchased from Jackson Laboratories at 6 weeks of age as a B6D2F1 stock (#100006) from a cross of C57BL/6 females with DBA/2 males. Upon arrival at our facility, animals were placed in individual vented cages in rooms with 12:12 light cycle and were fed (Purina Pro-Lab 5V75) *ad libitum*. When mice reached 8 weeks of age, they were randomized into treatment groups as described in Figure 1. All animal procedures were approved by MUSC IACUC.

### Mouse Treatment

Metformin hydrochloride (M) (Tokyo Chemical Industry, #M200925G) was utilized at 205 mg/kg. Hydroxychloroquine sulfate (C) (Tokyo Chemical Industry, #H13065G) was used at 100 mg/kg. Nelfinavir mesylate (N) (Agouron Pharmaceuticals, 625 mg tablets, #63010-027-70) was used at 250 mg/kg. Rapamycin (R) (LC Laboratories, #R50000100MG) was used at 2.24 mg/kg. Dasatinib (D) (LC Laboratories, #D3307500MG) was used at 10 mg/kg/day. Doxil (Reddy’s Laboratories Inc., 2 mg/ml, #43598-283-35) was used at 15 mg/kg. Microcrystalline cellulose, used in gavage vehicle control, was used at 226 mg/kg. Autophagy drugs (M,C,N,R,D) and microcrystalline cellulose were suspended in 50% PEG400 (Fisher Scientific, # NC9443499) in water (VWR, #L0201-0500) and administered by 20G oral gavage needles (Fisher Scientific, #NC1352689), daily, under 2% isoflurane anesthesia (Henry Schein, #1182097, given through vaporizer made by Paragon Medical, #M1200V) for less than 5 min. Doxil or vehicle control (0.9% sterile saline, Fisher Scientific, #50-843-140) were injected intraperitoneally using a 26G 12.7 mm needle (VWR, #305111) immediately after the gavage injection on day 8. Injection volumes were adjusted for mouse weight to maintain consistent mg/kg, with the maximal volume being 200 µl for a 30 g mouse, consistent with our prior efficacy studies (Delaney et al., 2015; Davis et al., 2016; Delaney et al., 2017).

To allow for sufficient time to dissect and process all tissues for pathology at endpoint, mice were staggered for treatment and euthanasia in four groups separated by 1 day each. At each euthanasia point, 1–2 mice from each treatment group were processed, allowing for all controls and experimental groups to be processed each day to reduce potential batch effects. Since food consumption habits may depend on the number of mice in a cage, only the days in which all five mice were present and all under treatment were quantified for food consumption. Food was weighed and replaced daily and the difference in weight quantified. A pilot was initially performed to observe if Doxil (15 mg/kg) was tolerated with or without five-drug COAST, using N = 2 mice. For whole-mouse weight measures (Figure 1B), these additional 2 mice are included in the analysis for vehicle control, five-drug COAST, Doxil, and five-drug COAST + Doxil. However, these two pilot mice are not included in subsequent figures as they were not assessed for full pathological examination. Individual data points per mouse are shown in all figures where available, to assist in data transparency and outlier interpretation (Ortell et al., 2019).

### Sample Collection

Twenty-four hours after final drug administration, mice were transferred to a veterinary pathology core facility to process mice for 1) organ dissection, weight, and formalin fixation (10% formalin for 24 h) for FFPE block creation, slide sectioning, and H&E staining, 2) blood biochemistry and complete blood count analysis and 3) partial spleen dissection (the other portion was used for histology) for flow cytometric quantitation of immune cells. Blood was collected *via* cardiac puncture after euthanasia.

### Biochemical Analysis

Blood samples were analyzed for liver and kidney biochemistry markers using vendor protocols for the VetScan VS2 instrument (Abaxis).

### Immune Cell Phenotyping

Immunophenotyping was performed by flow cytometry as detailed in our previous reports (Karumuthil-Melethil et al., 2008; Perez et al., 2008). Briefly, single cell suspensions of spleens were stained using different fluorochrome labelled antibodies against mouse CD4, CD8, CD19, CD11c. CD11b, and Foxp3 (Invitrogen). Staining for Foxp3 was done using intranuclear staining buffer kit from Invitrogen. The stained samples were acquired using FACSVerse instrument (BD Biosciences and the data was analyzed using FlowJo software (BD Biosciences). The frequencies of various immune cell populations among all spleen cells were quantified as B cells (CD19^+^), helper T cells (CD4^+^), cytotoxic T cells (CD8^+^) regulatory T cells (CD4^+^Foxp3^+^), monocytes (CD11b^+^) and dendritic cells (CD11c^+^). CD4^+^ population was gated for determining Foxp3^+^ cell (CD4^+^Foxp3^+^ regulatory T cells) frequencies. Samples stained using isotype control antibodies were used to gate for each specific population. Differences in numbers of mice used per group were due to processing accidents preventing accurate preparation or interpretation of data, and were each made on blinded samples (N = 1 vehicle control mis-processed, N = 2 Doxil controls mis-processed).

### Histological Assessment

Histological assessment of all organs was completed for five mice per group. Pathologist was blinded to treatment group at time of analysis. Samples were subsequently unblinded and the summary Table 1 was created. Note that uterus pathology is not herein reported since the mouse estrus cycle was not synchronized, precluding informative group comparisons.

**TABLE 1:** Veterinary Histological Pathology. Numbers represent the number of mice with noted phenotype, with a total of five mice per treatment group.

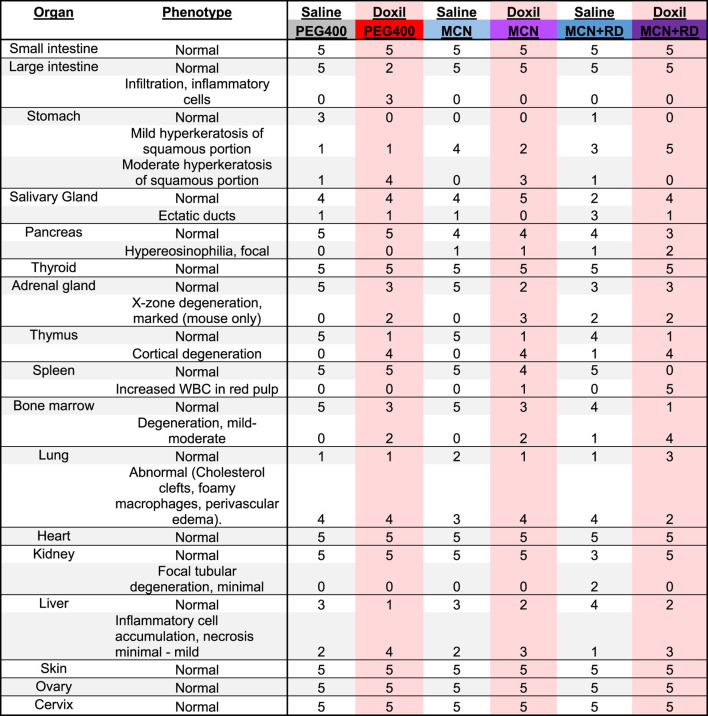

### Statistical Analysis

Two-tailed, Student’s *t*-tests were used to calculate statistical significance. *p* ≤ 0.05 was considered statistically significant. * is *p* ≤ 0.05 in all figures, with red asterisks comparing to the Doxil group and grey asterisks comparing to the vehicle control group. No statistics were performed on histological examinations, as a single-mouse observed pathology may be informative if these drugs are used in broad human populations.

## Results

### Experimental Setup to Combine COAST Autophagy Drugs With Doxil *in Vivo*

Our previous efficacy experiments showed complete tumor remission using a 15-days treatment of chloroquine phosphate (30 mg/kg), nelfinavir mesylate (250 mg/kg), rapamycin (2.24 mg/kg), dasatinib (4 mg/kg) and metformin (150 mg/kg) in 50% PEG400. However, hydroxychloroquine is more often utilized in human studies than chloroquine phosphate due to a modest reduction in side effect. Furthermore, dasatinib and metformin at the above mouse doses are slightly lower than what are known to be maximally tolerated in humans, using the FDA’s formula for drug dose conversion (Delaney et al., 2015). Rapamycin dose is also lower than some cancer trial doses, as this dose is known to extend lifespan in mice and better mimic the human dose which can improve rather than hinder immunological response (Mannick et al., 2014; Mannick et al., 2018). To best mimic what may represent a potentially efficacious, maximal dose of COAST and observe associated side effects in a preclinical setting, we opted to treat mice for 14 days using hydroxychloroquine (100 mg/kg), metformin (205 mg/kg), and nelfinavir mesylate (250 mg/kg) with or without the additional COAST drugs rapamycin (2.24 mg/kg) and dasatinib (10 mg/kg). Potential human trials would likely additionally combine COAST with a chemotherapy.

Since much of our previous data was in high-grade serous ovarian cancer, we opted to use Doxil, a second-line chemotherapy used in ovarian cancer composed of a peggylated liposomal form of doxorubicin, as our candidate combinatorial chemotherapy agent. Doxil was dosed once at 15 mg/kg in the middle of COAST therapy (Figure 1A). Doxil also served as our positive control for known systemic toxicity, albeit modestly reduced compared to doxorubicin (Bhinge et al., 2012; Tacar et al., 2013; Farhad et al., 2016). Our previous *in vitro* results suggest that autophagy drugs potentiate the effects of chemotherapy given concurrently, prior, or subsequent to chemotherapy (Delaney et al., 2017; Bowers et al., 2022a), but no previous data on combinatorial safety *in vivo* has been obtained. In this study, we randomized female mice, treated N = 5 mice in each of these six drug groups, and then euthanized mice for pathological examination by histochemistry, blood biochemistry, and flow cytometric analysis of spleen cells.

### Mouse Body-Weight

As initial measures of severe systemic toxicity, we performed weight measurements of mice during treatment. While some individual mice gained or lost weight, no single mouse surpassed >10% weight loss in any group examined (Figure 1B). No clear pattern of weight change was associated with Doxil or COAST drug groups. Compared to vehicle control, there was a statistically significant average weight loss in the presence of Doxil in the three-drug COAST group (4.6% loss, *p* ≤ 0.05), but not in the five-drug COAST group (0.6% gain).

We additionally examined whether mice consumed more or less food during therapy. Control mice consumed, on average, approximately 2.4 g/mouse/day (Figure 1C). The only significant difference from the vehicle control was again the five-drug COAST group, which consumed 2.9 g/mouse/day during treatment (*p* ≤ 0.05).

### Liver Biochemistry

**FIGURE 2:**
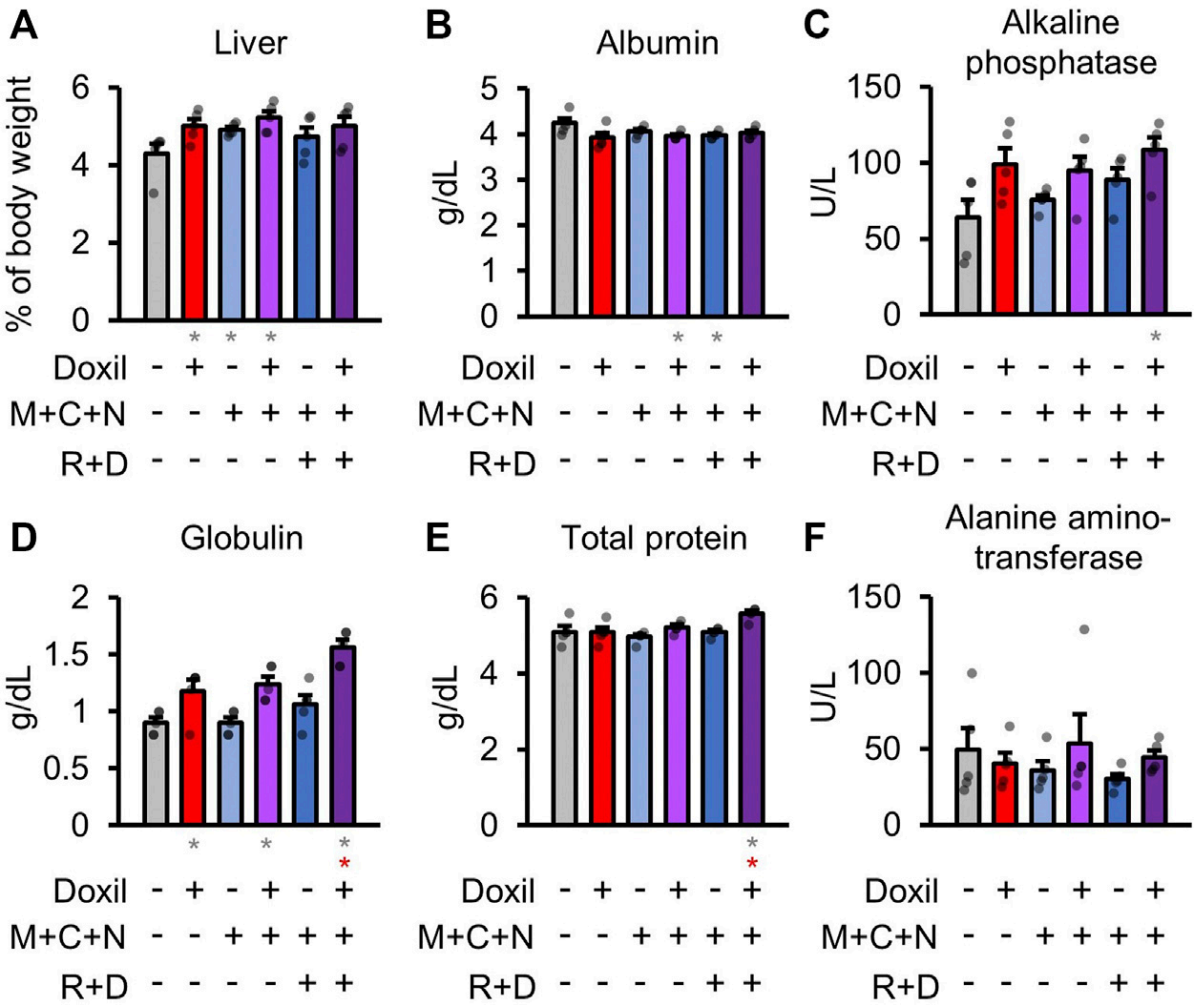
Liver weights and biochemistry. **(A)**. Liver weights, normalized to whole mouse weight. **(B–F)**. Blood biochemistry panel markers most related to liver function. Gray * indicates *p* ≤ 0.05 relative to vehicle control, red * indicates *p* ≤ 0.05 relative to Doxil.

Health of the liver was initially assessed through blood biochemistry markers of liver function and organ weight. Statistically significant (*p* ≤ 0.05) increases in liver weight were observed for the Doxil, MCN, and Doxil + MCN groups (Figure 2A). A modest but statistically significant decrease in blood albumin, indicative of possible liver or kidney dysfunction, was observed in the Doxil + MCN and MCN + RD groups (Figure 2B). Alkaline phosphatase activity trended toward an increase, indicative of possible liver dysfunction, in all Doxil groups, and was statistically significant relative to vehicle control (but not to Doxil alone) in the Doxil + MCN + RD group (Figure 2C). Globulin was significantly increased, indicative of inflammation (low levels, which were not observed, may indicate liver dysfunction), in all Doxil treated groups, with a further increase from Doxil in the Doxil + MCN + RD group (Figure 2D). Total protein was significantly increased in the Doxil + MCN + RD group, indicative of inflammation in the context of elevated globulin (Figure 2E). Alanine amino-transferase, another marker of liver damage, was not significantly altered in any group, and did not trend toward an increase (Figure 2F).

### Kidney Biochemistry

**FIGURE 3:**
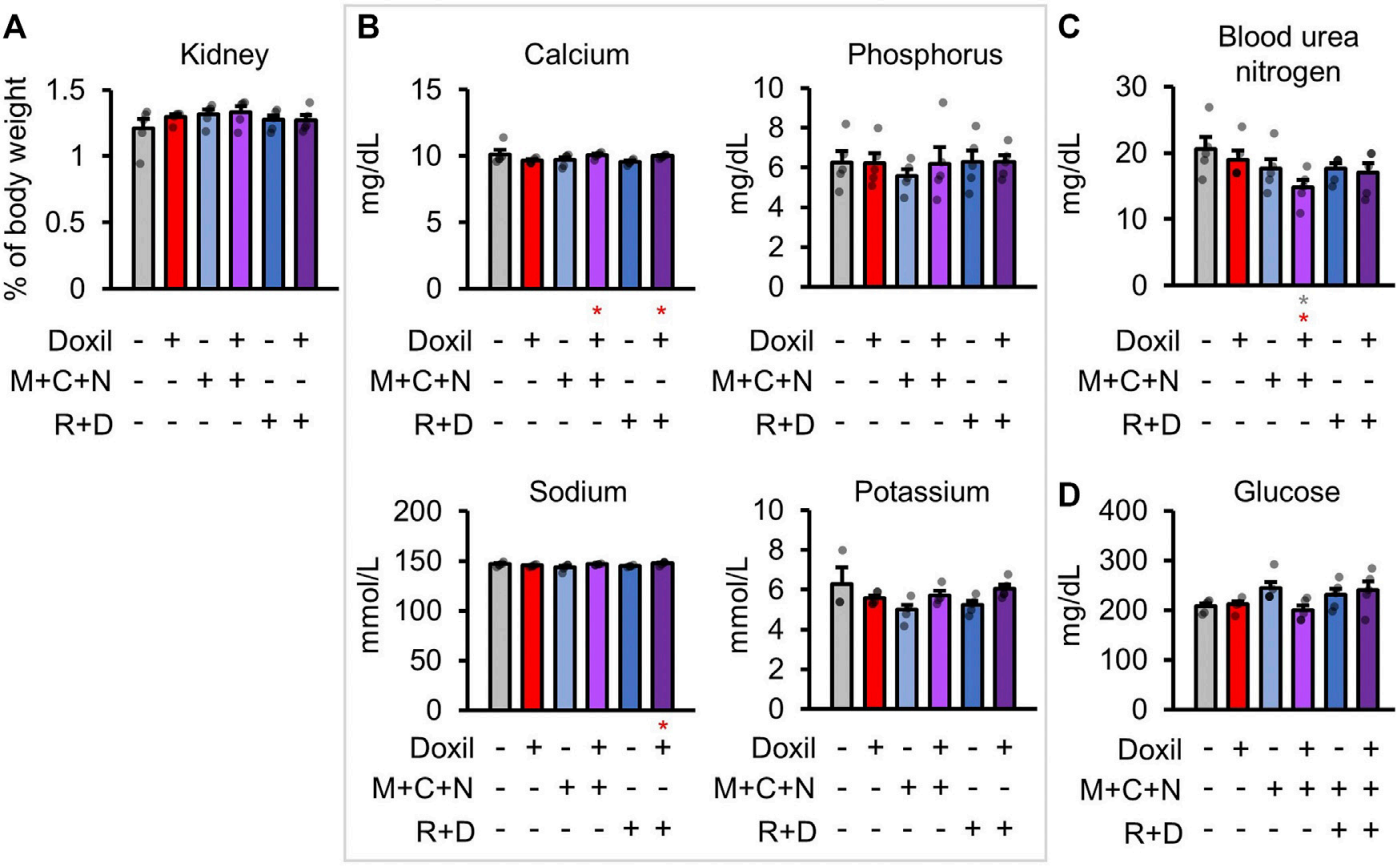
Kidney weights and biochemistry. **(A).** Kidney weights, normalized to whole mouse weight. **(B–D)**. Blood biochemistry panel markers most related to kidney function. Gray * indicates *p* ≤ 0.05 relative to vehicle control, red * indicates *p* ≤ 0.05 relative to Doxil.

Health of the kidney was initially assessed through blood biochemistry markers of kidney function and organ weight. There were no significant differences in kidney organ weight (Figure 3A). Kidneys are the central regulators of blood ion concentration. Calcium, phosphorus, sodium, and potassium levels were not significantly different from control samples (Figure 3B). A minor trend toward a decrease in calcium was observed in Doxil, which was significantly ameliorated in the Doxil + MCN and Doxil + MCN + RD groups (*p* < 0.05). Another amelioration was observed for sodium, with a trend toward lower levels in Doxil and a significant increase relative to Doxil in the Doxil + MCN + RD group (*p* < 0.05). Blood urea nitrogen (BUN), which may be indicative of kidney dysfunction if elevated, trended lower than control and was significantly lower than control and Doxil in the Doxil + MCN group (*p* < 0.05) (Figure 3C). Low BUN can be associated with elevated hydration, malnutrition, or liver disease. Blood glucose is carefully controlled with many factors including the liver and kidney. No significant differences in blood glucose were observed, although a trend toward increases was observed in COAST treatment groups (Figure 3D).

### Red Blood Cell Counts

**FIGURE 4:**
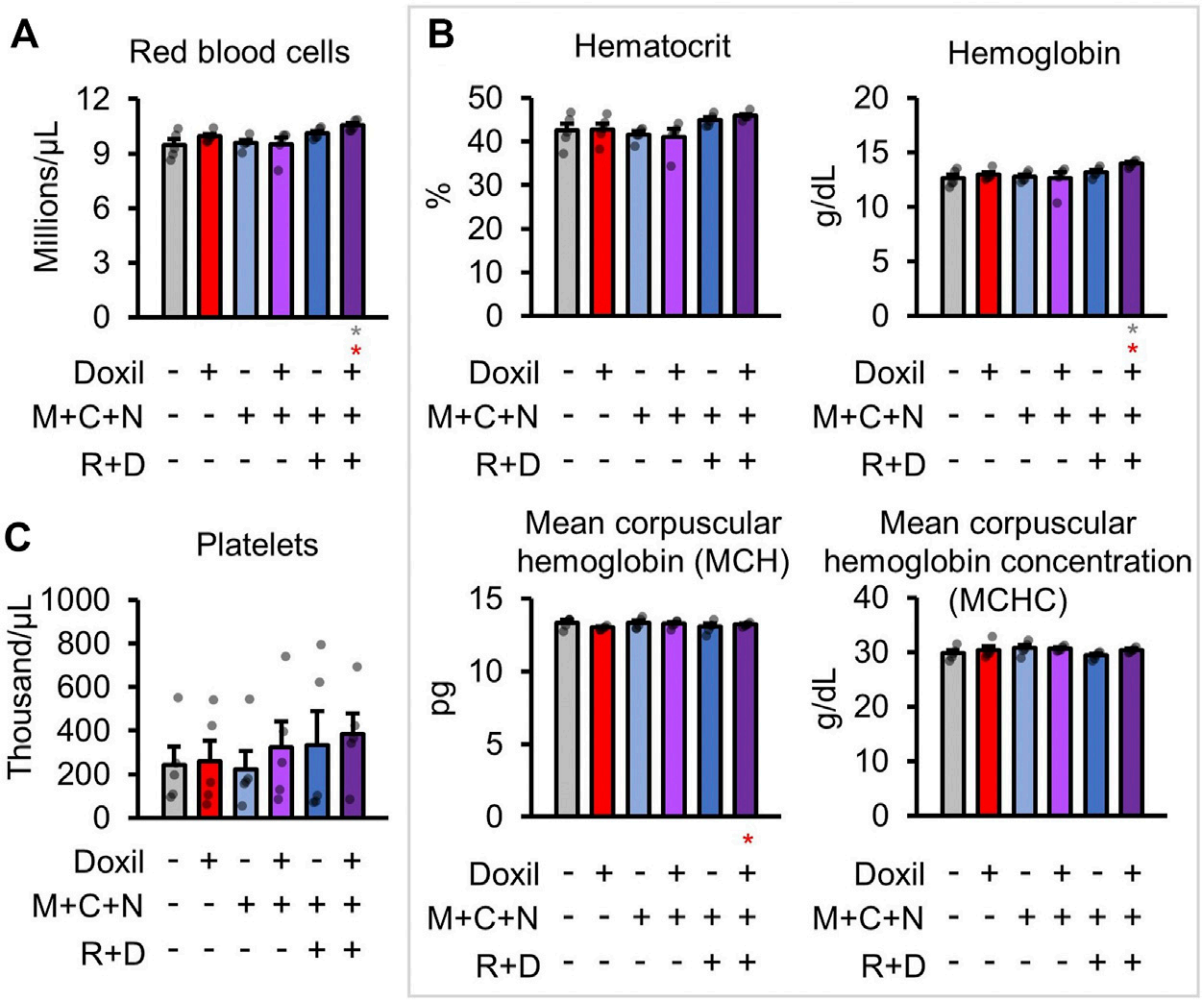
Red blood cell measures. **(A)**. Red blood cell raw counts. **(B)**. Blood biochemistry panel markers and calculations most related to red blood cell function. **(C)**. Platelet raw counts. Gray * indicates *p* ≤ 0.05 relative to vehicle control, red * indicates *p* ≤ 0.05 relative to Doxil.

Next, red blood cells (RBC) were measured. No decreases in red blood cell count were observed in any group (Figure 4A). An increase in RBC relative to control and Doxil was observed in the Doxil + MCN + RD group (*p* < 0.05). Measures of RBC parameters, including hematocrit, hemoglobin, mean corpuscular hemoglobin (MCH), mean corpuscular hemoglobin concentration (MCHC), did not show any changes except in the Doxil + MCN + RD group (Figure 4B). In Doxil + MCN + RD, hemoglobin and MCH were significantly increased, which could be indicative of effects of the drug on RBC precursors in hematopoietic tissue, hypoxia sensation or liver or kidney dysfunction.

**FIGURE 5:**
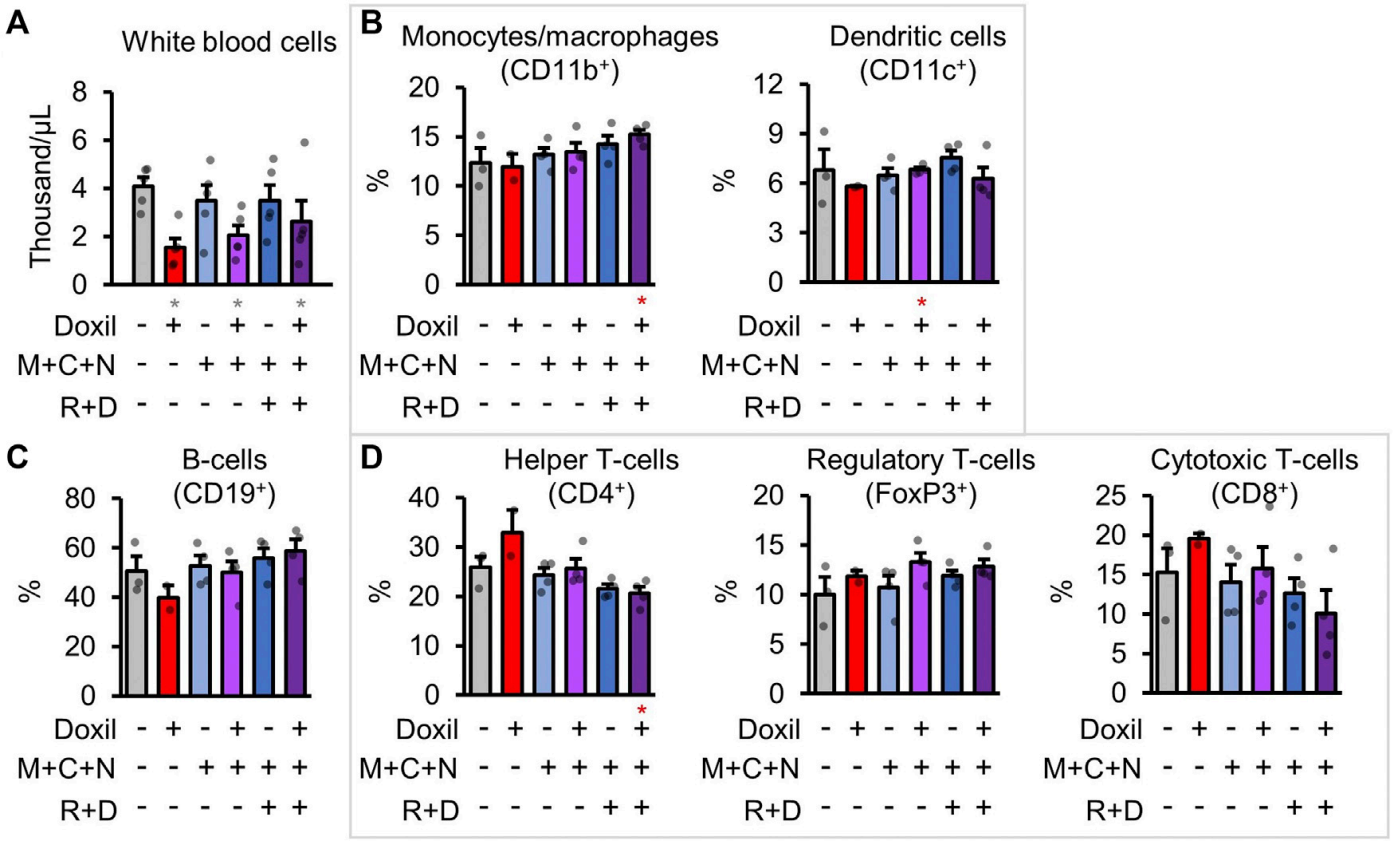
Immunologic blood cell quantitation. **(A)**. White blood cell raw counts. **(B–D)**. Flow cytometric measures of immune cell types, with select markers indicated in parentheses. Hierarchy is described in methods. Gray * indicates *p* ≤ 0.05 relative to vehicle control, red * indicates *p* ≤ 0.05 relative to Doxil.

White-blood cells (WBCs) were counted directly from blood samples. As expected, Doxil significantly decreased overall white blood cell counts in all groups (*p* < 0.05), with some trend toward amelioration of that decline in the COAST groups (Figure 5A). However, it is possible that the autophagy-modulating drugs could impact the proportion of different immune cell populations. To address this possibility, cells from a secondary lymphoid organ, spleens, were examined for the frequencies of various immune cell types by flow cytometry after staining using specific antibodies (see Methods). Note that absolute counts of cell lineages was not possible since the spleen was split for use in histology and flow cytometry. Doxil + MCN + RD contained proportionally more monocyte and macrophages (Figure 5B). Due to less observed variation, dendritic cells were significantly higher in Doxil + MCN cells than Doxil cells (*p* < 0.05) but were comparable to vehicle control cells (Figure 5B). B-cells were even between groups, with a trend toward a decrease in the Doxil group (Figure 5C). This decrease in B-cell proportion may be attributed to the observed trend toward increases in T-cell sub-populations in the Doxil group (Figure 5D). There was a decrease in helper T-cells in Doxil + MCN + RD relative to Doxil (*p* < 0.05) and a trend toward a decrease in cytotoxic T-cells in Doxil + MCN + RD, but interpretation of this percent change is limited since these groups trended toward more overall white blood cells in the peripheral blood than the Doxil group.

### Histopathology

**FIGURE 6:**
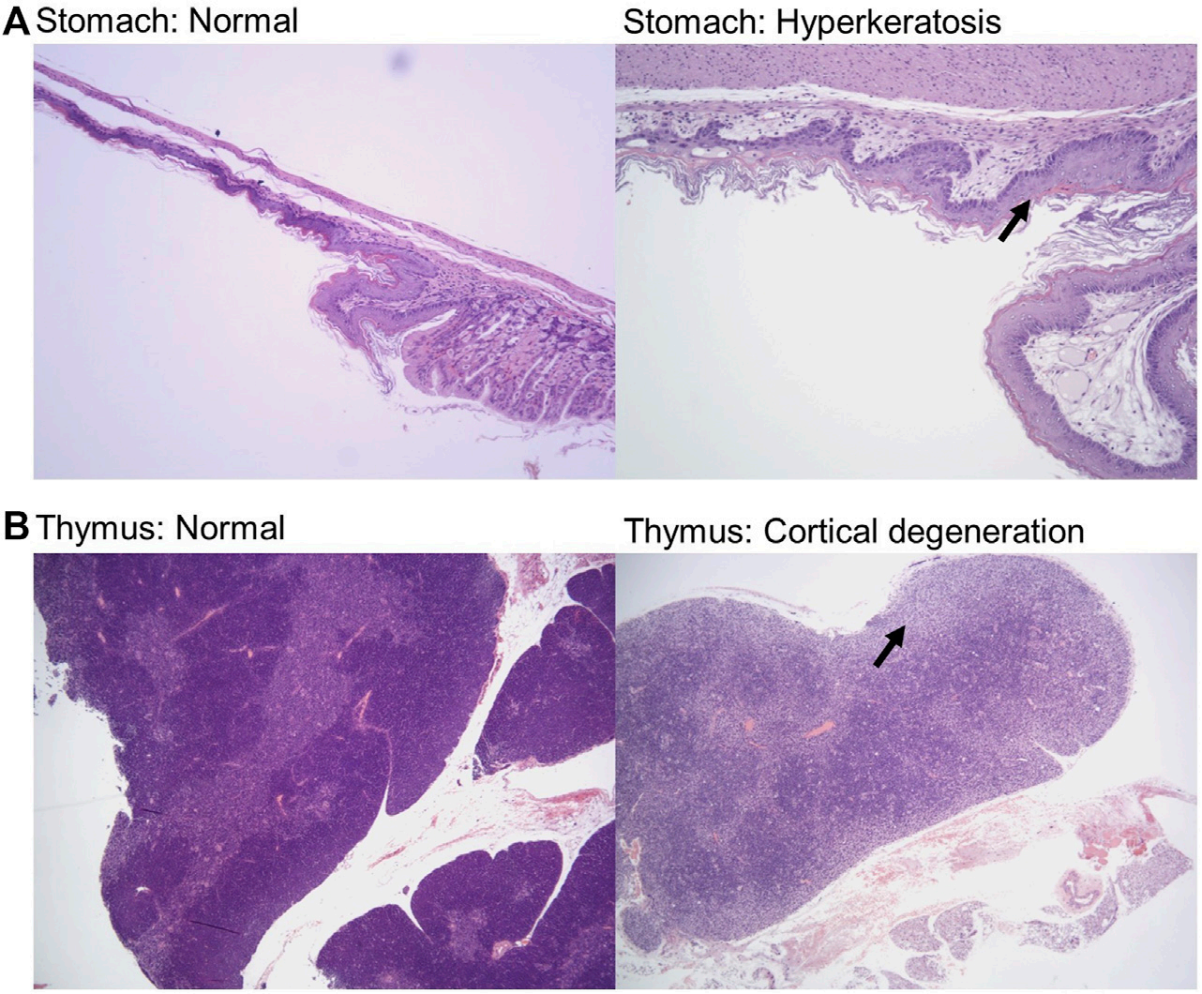
Histopathology representative images of stomach hyperkeratosis and thymus cortical degeneration. **(A)** H&E stained images of stomach sections from a vehicle control mouse (left panel) and a Doxil treated mouse (right panel). Images shown at ×10 magnification. Arrow highlights hyperkeratosis. **(B)** H&E stained images of thymus sections from a vehicle control mouse (left panel) and a Doxil treated mouse (right panel). Images shown at ×4 magnification. Arrow highlights cortical degeneration.

Samples were fixed, embedded, sectioned, stained for hematoxylin and eosin (H&E), and analyzed by a veterinary pathologist in a blinded fashion. Here, we report noted aberrations in Table 1. We observed that in some cases, control groups were abnormal due to pathogenic effects of daily oral gavage of viscous solutions into mice (e.g, abnormal lung histology). As expected, Doxil caused observable toxicity to immune-system related tissues such as the spleen, thymus, and bone marrow. MCN and MCN + RD associations were more complex, with MCN sometimes exhibiting possible toxicity while MCN + RD did not. No organ had a clear consistent pathology with MCN or MCN + RD treatment relative to Doxil or vehicle controls. Hyperkeratosis of the stomach (Figure 6A) and cortical degeneration of the thymus (Figure 6B) were the most consistent changes and were associated with Doxil administration.

## Discussion

We here report a pre-clinical study of the effects of complex autophagy drug combinations with or without the chemotherapy Doxil. Complete blood counts, blood biochemistry, and veterinary pathology are reported alongside gross weight measurements. These findings are intended to inform future clinical trial design of autophagy-targeting drugs, particularly those included in this study: metformin, hydroxychloroquine, nelfinavir mesylate, rapamycin, and dasatinib. These results follow our previous research indicating simultaneous induction of autophagy (LC3-II levels), prohibition of autophagosome clearance (p62 and intracellular vesicle accumulation), and endoplasmic reticulum stress response (by GRP78) *in vitro* and *in vivo* using COAST drugs (Delaney et al., 2015; Davis et al., 2016; Delaney et al., 2017).

Perhaps the most surprising find was the limited observable toxicity of a high-order autophagy drug combination: MCN + RD. The only consistently noted aberrations common in MCN and MCN + RD groups were an elevation of glucose and histological stomach aberrations, which should be appropriately monitored in clinical trials. The stomach aberrations were primarily found in the squamous portion of the stomach to which there is no human comparison. The lack of a similar anatomic region in humans does not mitigate the need for monitoring changes in those receiving this combination of treatment agents. The lack of other toxicities from 14 daily treatments is in stark contrast to what was observed from a single dose of Doxil. These results, taken together, suggest the toxicity of the autophagy modulating drugs used here may be reduced relative to standard cytotoxic chemotherapy treatment of cancer. This finding is consistent with our previous studies in mice which were also inoculated with cancer (Delaney et al., 2015; Davis et al., 2016; Delaney et al., 2017), however, it was previously unclear if mice were healthier simply due to reduced tumor burden. Since this current study was performed in the absence of cancer, toxicity or lack of toxicity is better attributed to the drugs alone.

One main purpose of this study was to determine if there was a severe interaction of Doxil, a form of doxorubicin, with the autophagy drugs in COAST. Previous literature suggests doxorubicin incurs cardiotoxicity in part due to dysregulation of autophagy (Dirks-Naylor, 2013; Sishi et al., 2013; Koleini and Kardami, 2017). Furthermore, since doxorubicin is a DNA intercalating agent and requires efficient DNA repair pathways, an interaction with COAST drugs was possible since autophagy is well-established to be upregulated during DNA repair (Rodriguez-Rocha et al., 2011; Czarny et al., 2015; Eliopoulos et al., 2016). While our small sample size may preclude the observation of some interactions, we were somewhat surprised to see a trend toward amelioration of Doxil toxicity in some of the measures performed. For example, while white blood cell count was decreased by 67% in the Doxil treated group, it was only decreased by 49% in the Doxil + MCN group and 36% in the Doxil + MCN + RD group. While the percentage of cytotoxic T-cells decreased in the spleen of Doxil + MCN + RD group, the total number of cytotoxic T-cells was unlikely reduced relative to the Doxil group, given the trend toward white blood cell count increase in the peripheral blood. Our methods did not detect cardiotoxicity in the Doxil group and accordingly did not find any cardiotoxicity in Doxil + MCN or Doxil + MCN + RD.

The most concerning interactions between Doxil and COAST were found in the Doxil + MCN + RD group. These included elevated blood globulin and hemoglobin. Elevated globulin may be associated with systemic inflammation. However, the current study does not specifically demonstrate inflammation. Doxil decreased WBC count, whereas Doxil + MCN + RD only trended toward a WBC increase compared to Doxil. This could equivalently be explained by an amelioration of WBC depletion. Future studies will be needed to assess the functionality of immune cells by examining for their pro-inflammatory and immune regulatory cytokine expression profiles. Disrupted autophagy is known to affect inflammation, particularly in CD11c^+^ and CD45^+^ cells (Ilyas et al., 2019; Galle-Treger et al., 2022). Total cell numbers and the frequencies of immune cells populations in the peripheral blood and lymphoid organs will inform if the therapy has an impact on immune cell mobilization. Taken together, inflammation should be monitored in a clinical trial investigating the combination of Doxil and MCN + RD drugs.

Limitations of the study included a limited sample size of most often N = 5 per group, an all-female mouse population, mouse-specific organ differences (the squamous portion of the stomach and the X-zone of the adrenal gland does not exist in humans), and a drug administration period limited to 2 weeks. While the adrenal X-zone does not exist in humans, it is endocrine dependent, and as such may be relevant to humans when a difference is seen among treatment groups. Since all mice were euthanized after the last dose, it is unclear if any observed pathology would self-correct after a period of healing.

## Conclusion

Fourteen doses of five combined autophagy drugs were broadly less toxic than a single dose of the chemotherapeutic Doxil. Two COAST drugs, hydroxychloroquine and nelfinavir, prohibit autophagic flux. Autophagic flux is established to be required to survive the insults of chemotherapy in cancer cells both *in vitro* and *in vivo*. Clinical trials incorporating autophagy disruption with chemotherapy may consider incorporation of the drugs used here (metformin, hydroxychloroquine, nelfinavir mesylate, rapamycin, and dasatinib) as candidates with well-studied toxicity profiles in the mouse model studied herein.

## Data Availability

The original contributions presented in the study are included in the article/supplementary material, further inquiries can be directed to the corresponding author.

## Abbreviations

C, Hydroxychloroquine; COAST, Combination Of Autophagy Selective Therapeutics; D, Dasatinib; M, Metformin; N, Nelfinavir mesylate; R, Rapamycin; WBC, White blood cell.
